# Type-I heavy tailed family with applications in medicine, engineering and insurance

**DOI:** 10.1371/journal.pone.0237462

**Published:** 2020-08-27

**Authors:** Wei Zhao, Saima K. Khosa, Zubair Ahmad, Muhammad Aslam, Ahmed Z. Afify

**Affiliations:** 1 School of Economics, Central University of Finance and Economics, Beijing, China; 2 Department of Statistics, Bahauddin Zakariya University, Multan, Pakistan; 3 Department of Statistics, Yazd University, Yazd, Iran; 4 Department of Statistics, Faculty of Science, King Abdulaziz University, Jeddah, Saudi Arabia; 5 Department of Statistics, Mathematics and Insurance, Benha University, Benha, Egypt; Tongii University, CHINA

## Abstract

In the present study, a new class of heavy tailed distributions using the T-*X* family approach is introduced. The proposed family is called type-I heavy tailed family. A special model of the proposed class, named Type-I Heavy Tailed Weibull (TI-HTW) model is studied in detail. We adopt the approach of maximum likelihood estimation for estimating its parameters, and assess the maximum likelihood performance based on biases and mean squared errors via a Monte Carlo simulation framework. Actuarial quantities such as value at risk and tail value at risk are derived. A simulation study for these actuarial measures is conducted, proving that the proposed TI-HTW is a heavy-tailed model. Finally, we provide a comparative study to illustrate the proposed method by analyzing three real data sets from different disciplines such as reliability engineering, bio-medical and financial sciences. The analytical results of the new TI-HTW model are compared with the Weibull and some other non-nested distributions. The Baysesian analysis is discussed to measure the model complexity based on the deviance information criterion.

## Introduction

In many practical situations, such as financial sciences, reliability engineering and bio-medial sciences, data are usually positive, and their distribution is unimodal hump shaped and extreme values yielding heavier tails than the classical models. For example, in health science research, (a) the medical expenditures that exceed a given treshould [1] and (b) the lenght of stay in hospitals [2, 3], present highly skewed, heavy tailed data, for which the standard classical distributions and simple variable transformation are insufficient to provide an adequate fit to such data. In reliability engineering, the interest most often lies in the occurrence of rather exceptional events which are associated with the tail part of a statistical distribution. For example, the earthquakes, tsunamis, hurricanes, electrical or power massive failures etc., are some examples of such type of rare and extreme events [4]. All aforementioned events and the rate at which they happens, are associated with the heaviness of the tail and shape of distributions. In financial and risk management problems, one of the important tasks is to predict accurately the losses that occurs with a high fiscal value. Underestimation of the probability of these losses leads to severe operational risk like underestimating the bankruptcy and premium, among others. In such circumstances, the models with heavy-tailed are the best candidate that provides plausibly the best fit for the right tail, see [5].

According to [6], a distribution is said to be heavy-tailed, if the right tail probabilities are heavier than the exponential distribution, that is, its survival function (sf) satisfies

$$\begin{array}{c} \underset{x\to \infty }{\text{lim}}\frac{1-F(x)}{e^{-px}}=\infty , \end{array}$$

for all *p* > 0; see [7]. The right tail of a model is an important issue in a number of contexts, particularly, pertaining to the insurance problems, where it shows the total impact of insurance losses, and in risk theory, where it is associated with the extreme value theory.

As we discussed above, on a number of occasions, real data sets show a behavior with extreme values producing tails which are heavier than those of classical well-known statistical distributions. In such cases, the utilization of the standard distributions may be not be a good choice to apply; see [8]. They performed an empirical study of distributions using exploratory data analysis and other empirical approaches to estimate the risk. They rejected the idea of using exponential, gamma and Weibull distributions due to their poor results and pointed out that one would need to use a model that is flexible enough in its structure.

These results motivated the researchers to look for more flexible models providing the best fit with greater accuracy in modeling data. In this regard, a number of approaches for extending and generalizing heavy-tailed distributions have been introduced. The new developments have been made through many different approaches such as (i) transformation of variables, (ii) composition of two or more models, (iii) compounding of models, and finally (iv) finite mixture of models.

Recent the study of [9] showed that skewed student *t* model and skewed-normal model are the best competitors as the skewed distributions adjust right-skewness and high kurtosis; for further detail see [10]. Financial risks and the insurance losses take positive values on the real line and consequently these skew models may not be suitable choice. In such situations, the transformation of variables, particularly the exponential transformation, has proven to be substantial because these distributions are defined on R. Furthermore, [11] showed that the transformation approach is simple to use but most often the inference as well as derivation of the other statistical properties become complicated.

Another promising approach for obtaining new flexible heavy-tailed families of distributions, which might provides reasonably the best fit for heavy-tailed losses, is the composition approach; see [12]. However, it must be noted, the new distributions obtained via the composition approach usually involve three or more parameters causing difficulties in the estimation and computational processes.

Another prominent approach is compounding of distributions to cater data modeling with unimodality, right-skewness and heavy tails [13]. However, the density function obtained via this approach may not always have a closed form expression which makes the estimation more complicated as shown in [14].

Finite mixture models represent a further approach to define very flexible distributions which are also able to capture, for instance, multimodality of the underlying distribution [15]. The price to pay for this greater flexibility is a more complicated and computationally challenging inference.

We carry on this branch of distribution theory, and propose a new family of heavy-tailed distributions via T-*X* family technique. The proposed class is very flexible and provides the best fit for the considered heavy-tailed insurance data.

The rest of work done in study is arranged in the following sections: the proposed family is discussed in Section 2. A sub-case of the proposed class is introduced and the shapes of its density and hazard rate functions are sketched in Section 3. Statistical properties of the new family are obtained in Section 4. The expressions for the maximum likelihood estimators are derived in Section 5. In the same section, a Monte Carlo simulation study is presented. The actuarial measures are derived in Section 6. In the same section, a simulation study based on these measures is also provided. Practical applications are discussed in Section 7. Finally, the article is concluded in Section 8.

## Proposed method

This section offers the genesis of the proposed method. Recently, [16] proposed the T-*X* family method that is specified by the cumulative distribution function (cdf)

$$\begin{array}{c} G(x)=\int_{a_{1}}^{K[F(x;\xi )]}v(t)\;\;dt,\;\;\;\;\;\;x\in R, \end{array}$$ (1)

where *K*[*F*(*x*; *ξ*)] fulfills certain conditions; see [16]. The probability density function (pdf) corresponding to Eq (1) is

$$\begin{array}{c} g(x)=\{\frac{\partial }{\partial x}K[F(x;\xi )]\}\;\;v\{K[F(x;\xi )]\},\;x\in R. \end{array}$$

Deploying the T-*X* approach, a good deal of new families of statistical models have been proposed in the literature; see [17–20] and [21]. Let *T* ∼ exp(1), then its cdf is given by

$$\begin{array}{c} V(t)=1-e^{-t},\;t\geq 0. \end{array}$$ (2)

Corresponding to expression (), the density function is

$$\begin{array}{c} v(t)=e^{-t},\;t>0. \end{array}$$ (3)

If *v*(*t*) follows Eq (3) and setting $K[F(x;\xi )]=-\text{log}(\frac{1-F(x;\xi )}{1-(1-\theta )F(x;\xi )}{)}^{\theta }$ in Eq (1), the cdf of the type-I heavy-tailed (TI-HT) family follows as

$$\begin{array}{c} G(x;\theta ,\xi )=1-(\frac{1-F(x;\xi )}{1-(1-\theta )F(x;\xi )}{)}^{\theta },\;\theta >0,\;x\in R, \end{array}$$ (4)

where *F*(*x*; *ξ*) is the baseline distribution function which may depend on ξ∈R. Form Eq (4), we can see that *G*(*x*; *θ*, *ξ*) = *F*(*x*; *ξ*) for *θ* = 1.

Some key motivations of the proposed TI-HT method are the following: (i) An easy and convenient approach to modify the existing models, (ii) to improve the flexibility of the available models in the literature, (iii) to introduce a generalized form of existing models with closed expression for their distribution functions, (iv) to avail the best fit to real-world data as compared to other models with fewer parameters, same number of parameters and higher number of parameters and, (v) to provide an adequate fit to the heavy-tailed data in applied fields such as reliability engineering, medical and financial sciences and, other related fields.

The pdf associated to Eq (4) is

$$g(x;\theta ,\xi )=\frac{\theta^{2}f(x;\xi ){\left\{1-F(x;\xi )\right\}}^{\theta -1}}{{\left\{1-(1-\theta )F(x;\xi )\right\}}^{\theta +1}},\;x\in \mathbb{R}.$$ (5)

We concentrate our focus to a special sub-case of the new family, called type-I heavy-tailed Weibull (TI-HTW) distribution. Finally, we direct our attention to the results related to the TI-HTW model with real life data in three different disciplines. The first data set is taken from bio-medical field and the results of the TI-HTW model is compared to five other competitor distributions including (a) two-parameter Weibull distribution and (b) three-parameter models such as alpha power transformed Weibull (APTW), Marshall-Olkin Weibull (MOW), transmuted Weibull (TW) and modified Weibull (MW) distributions. The second data set is taken from reliability engineering and the comparison of the new model is made with three other well-known distributions such as (a) the three-parameter extended alpha power transformed Weibull (Ex-APTW) and (b) four-parameter Kumaraswamy Weibull (Ku-W) and beta Weibull (BW) distributions. The third data set is taken from financial sciences and the results of the proposed model is compared with Weibull and other heavy-tailed models including Lomax and Burr-XII distributions.

## Sub-model description

In the following section, we introduce the genesis of the TI-HTW distribution and discuss its special cases.

### Type-I heavy tailed Weibull distributionxs

Consider the cdf $F(x;\xi )=1-e^{-\gamma x^{\alpha }},\;x\geq 0$, and pdf $f(x;\xi )=\alpha \gamma x^{\alpha -1}e^{-\gamma x^{\alpha }}$, where *ξ* = (*α*, *γ*), of the two-parameter Weibull distribution with shape parameter *α* > 0 and scale parameter *γ* > 0. Then, cdf of the TI-HTW model is defined by

$$\begin{array}{c} G(x;\theta ,\xi )=1-(\frac{e^{-\gamma x^{\alpha }}}{1-(1-\theta )(1-e^{-\gamma x^{\alpha }})}{)}^{\theta },\;x>0,\;\;\alpha ,\theta ,\gamma >0. \end{array}$$ (6)

The pdf of the TI-HTW model is

$$g(x;\theta ,\xi )=\frac{\alpha \theta^{2}\gamma x^{\alpha -1}e^{-\theta \gamma x^{\alpha }}}{{\left\{1-(1-\theta )(1-e^{-\gamma x^{\alpha }})\right\}}^{\theta +1}},\;x>0.$$ (7)

**Fig 1:**
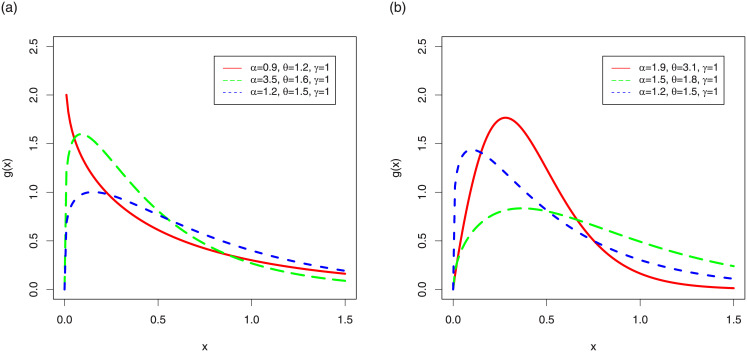
Pdf plots of the TI-HTW distribution.

**Fig 2:**
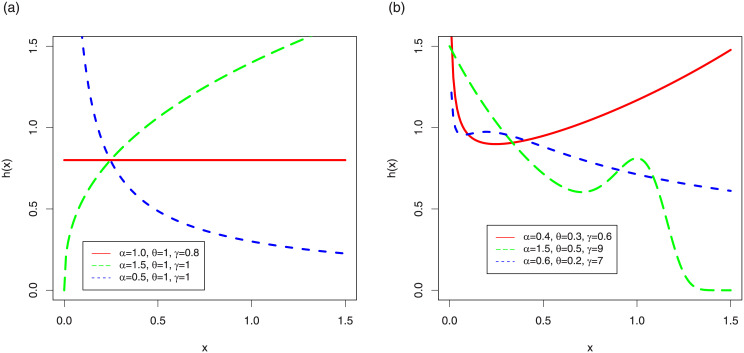
hrf plots of the TI-HTW distribution.

Plots for the pdf of the TI-HTW are sketched in Fig 1, whereas the hrf plots of TI-HTW are showed in Fig 2.

### Special cases of the TI-HTW distribution

Let *X* follows the TI-HTW model with parameters (*α*, *θ*, *γ*). Then *X* reduces to

1. Weibull model with parameters *α* and *γ*, with *θ* = 1.
2. One parameter Weibull model with parameter *α*, with *θ* = *γ* = 1.
3. Exponential with parameter *γ*, with *θ* = *α* = 1.
4. Rayleigh distribution with parameter *γ*, with *θ* = 1 and *α* = 2.
5. One parameter TI-HTW distribution with parameters *α* and *θ*, with *γ* = 1. (New)
6. Type-I Heavy Tailed Exponential (TI-HTE) with parameters *θ* and *γ*, with *α* = 1. (New)
7. Type-I Heavy Tailed Rayleigh (TI-HTR) with parameters *θ* and *γ*, with *α* = 2. (New)

## Statistical properties

In the following subsections, we study some statistical properties of the TI-HT distributions including the quantile function (qf), *r*^*th*^ moment and moment generating function.

### Quantile function

The qf of the TI-HT distributions is

x=Q(u)=G−1(u)=F−1{(1−u)1θ−1(1−θ)(1−u)1θ−1}, (8)

where *u* ∈ (0, 1). From expression Eq (8), we can see that the proposed model has closed form solution of the qf which makes it easier to generate random numbers for any sub-case of the TI-HT family.

### Moments

This sub-section deals with the derivation of *rth* moment of the TI-HT distributions. The *rth* moment of the TI-HT distributions is derived as

$$\begin{array}{c} \mu_{r}^{\prime }=\int_{-\infty }^{\infty }x^{r}g(x;\theta ,\xi )dx. \end{array}$$ (9)

Using Eq (5) in Eq (9), we have

$$\mu_{r}^{\prime }=\int_{-\infty }^{\infty }x^{r}\frac{\theta^{2}f(x;\xi ){\left\{1-F(x;\xi )\right\}}^{\theta -1}}{{\left\{1-(1-\theta )F(x;\xi )\right\}}^{\theta +1}}dx.$$ (10)

Using the expansion (https://math.stackexchange.com/questions/1624974/series-expansion-1-1-xn)

$$\begin{array}{c} \frac{1}{{\left(1-x\right)}^{n}}=\sum_{i=0}^{\infty }(\begin{array}{c} i+n-1\\ n-1 \end{array})x^{i}. \end{array}$$ (11)

Using *x* = (1 − *θ*)*F*(*x*; *ξ*) and *n* = *θ* + 1 in Eq (11), we get

$$\begin{array}{c} \frac{1}{{\left(1-(1-\theta )F(x;\xi )\right)}^{\theta +1}}=\sum_{i=0}^{\infty }(\begin{array}{c} i+\theta \\ \theta \end{array})(1-\theta {)}^{i}F(x;\xi {)}^{i}. \end{array}$$ (12)

Also using the series representation

$$\begin{array}{c} (1-y{)}^{m}=\sum_{j=0}^{m}(-1{)}^{j}(\begin{array}{c} m\\ j \end{array})x^{j}. \end{array}$$ (13)

Using *y* = *F*(*x*; *ξ*) and *m* = *θ* − 1 in Eq (13), we get

$$\begin{array}{c} {\left(1-F(x;\xi )\right)}^{\theta -1}=\sum_{j=0}^{\theta -1}(\begin{array}{c} \theta -1\\ j \end{array})(-1{)}^{j}F(x;\xi {)}^{j}. \end{array}$$ (14)

Using Eqs (12) and (14) in Eq (10), we have

$$\begin{array}{c} \mu_{r}^{\prime }=\theta^{2}\sum_{i=0}^{\infty }\sum_{j=0}^{\theta -1}(\begin{array}{c} \theta -1\\ j \end{array})(\begin{array}{c} i+\theta \\ \theta \end{array})(-1{)}^{j}(1-\theta {)}^{i}\kappa_{r,i+j}, \end{array}$$ (15)

where $\kappa_{r,i+j}=\int_{-\infty }^{\infty }x^{r}f(x;\xi )F(x;\xi {)}^{i+j}dx.$

For some pre-defined parameters values, numerical results for the descriptive measures (mean, variance, skewness and kurtosis of the TI-HTW mode are given in Tables 1 and 2.

**Table 1:** Descriptive measures of TI-HTW distribution for *α* = 0.9, *γ* = 1 and different values of *θ*.

*θ*	Mean	Variance	Skewness	Kurtosis
0.9	4.818964	126.9306	4.543845	27.37200
1.3	2.589755	48.53393	6.914864	65.00105
1.7	1.507111	17.71164	9.952248	146.0455
2.1	0.9776169	6.682181	13.02368	286.1895
2.4	0.753041	3.398202	14.67395	416.1977
2.8	0.5675229	1.525924	15.22652	557.1121

**Table 2:** Descriptive measures of TI-HTW distribution for *θ* = 0.5, *γ* = 1 and different values of *α*.

*α*	Mean	Variance	Skewness	Kurtosis
0.7	8.682646	308.9903	2.883661	11.42845
1.1	7.7619	232.2335	3.294275	14.82279
1.5	6.040198	150.1885	4.104262	22.65465
2.5	3.181183	40.31138	7.186336	72.47317
4.5	1.924026	7.084400	11.84863	248.0023
4.5	1.664518	3.115981	12.95318	363.5577

**Fig 3:**
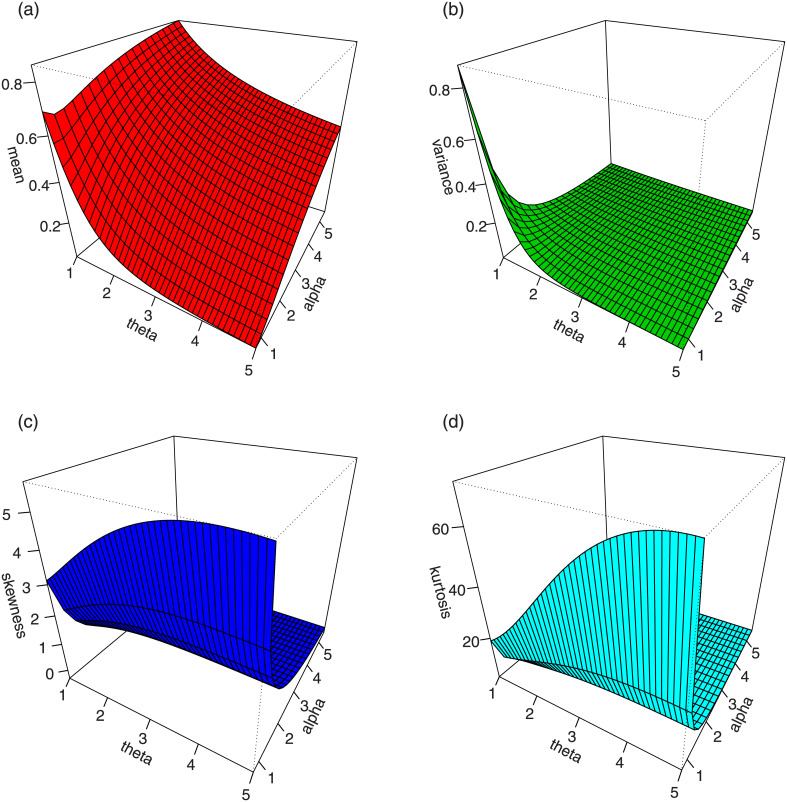
Plots for the mean, variance, skewness and kurtosis of the TI-HTW distribution.

**Fig 4:**
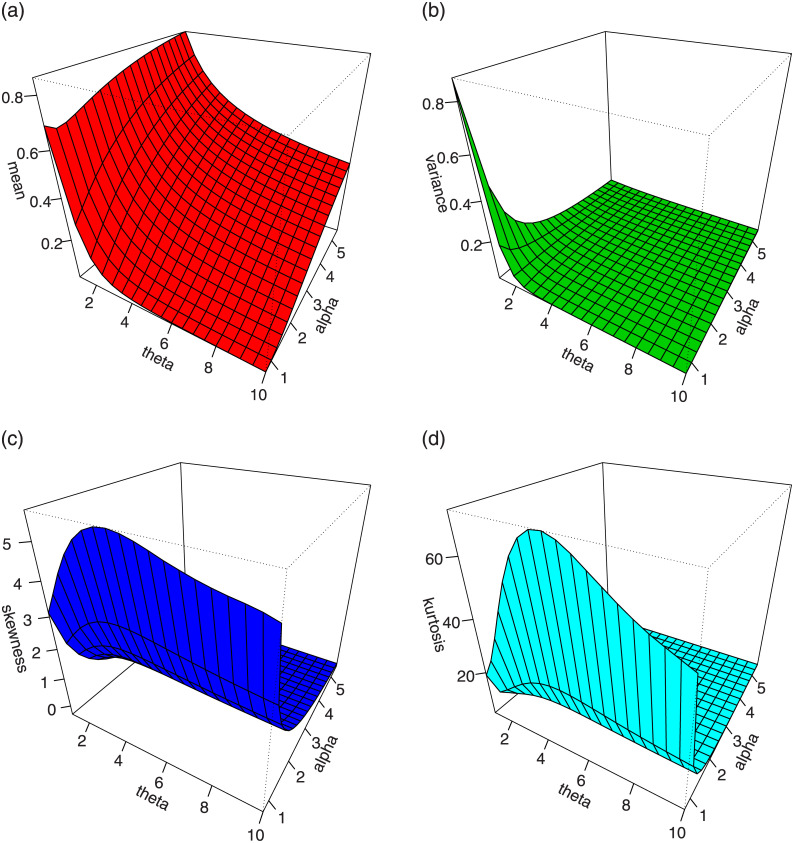
Plots for the mean, variance, skewness and kurtosis of the TI-HTW distribution.

For *γ* = 1.5 and different values *α* and *θ*, plots for the mean, variance, skewness and kurtosis of the TI-HTW distribution are displayed in Figs 3 and 4.

The moment generating function (mgf) of the TI-HT random variable *X*, say *M*_*X*_ (*t*), is derived as

$$\begin{array}{c} M_{X}(t)=\sum_{r=0}^{\infty }\frac{t^{r}}{r!}\mu_{r}^{\prime }. \end{array}$$ (16)

Using Eq (15) in Eq (16), we get the mgf of the TI-HT distributions.

## Estimation and simulation study

In the following section, we obtain the maximum likelihood estimators (MLEs) of the parameters of the proposed family. Furthermore, we conduct a Monte Carlo simulation study to assess the behavior of these estimators.

### Maximum likelihood estimation

Numerous approaches for estimating the un-known parameters have been suggested to obtain the estimates of the parameters. Among them, the maximum likelihood (ML) approach is the most prominent and frequently used method. The estimators obtained via this approach possess useful properties and can be utilized for constructing the confidence interval and other statistical tests. The normal approximation of the MLEs can easily be treated either numerically or analytically. For more details about maximum likelihood estimation, we refer to [22, 23]. In this sub-section, we adopt the ML approach for estimating the parameters of TI-HT family. Suppose *X*_1_, *X*_2_,…, *X*_*n*_ form an observed sample taken randomly from the TI-HT family with pdf (5). The corresponding log-likelihood function to (5) is

$$\begin{array}{c} \begin{array}{c} \ell (\Theta )=2n\;\text{log}\;\theta +\sum_{i=1}^{n}\;\text{log}\;f(x_{i};\xi )+(\theta -1)\sum_{i=1}^{n}\;\text{log}\;\{1-F(x_{i};\xi )\}\\ \;-(\theta +1)\sum_{i=1}^{n}\;\text{log}\;\{1-(1-\theta )F(x;\xi )\}, \end{array} \end{array}$$ (17)

where Θ = (*α*, *γ*, *θ*)^*T*^. The computer software such as ASS (PROC UNMIXED) can be used to maximize the log-likelihood function directly or via differentiating Eq (17). The partial derivatives of Eq (17) are given by

$$\begin{array}{c} \begin{array}{c} \frac{\partial \ell (\Theta )}{\partial \theta }=\frac{2n}{\theta }+\sum_{i=1}^{n}\text{log}\{1-F(x_{i};\xi )\}-\sum_{i=1}^{n}\text{log}\{1-(1-\theta )F(x_{i};\xi )\}\\ \;-(\theta +1)\sum_{i=1}^{n}\frac{F(x_{i};\xi )}{\left\{1-(1-\theta )F(x_{i};\xi )\right\}} \end{array} \end{array}$$ (18)

and

$$\begin{array}{ll} \frac{\partial l(\Theta )}{\partial \xi } & =\Sigma_{i=1}^{n}\frac{\partial f(x_{i};\xi )/\partial \xi }{\partial f(x_{i};\xi )}-(\theta -1)\Sigma_{i=1}^{n}\frac{\partial F(x_{i};\xi )/\partial \xi }{\left\{1-F(x_{i};\xi )\right\}}\\ & \text{+}(\theta +1)\Sigma_{i=1}^{n}\frac{(1-\theta )\partial F(x_{i};\xi )/\partial \xi }{\left\{1-(1-\theta )F(x_{i};\xi )\right\}}. \end{array}$$ (19)

Equating the nonlinear system of equations $\frac{\partial \ell (\Theta )}{\partial \theta }$ and $\frac{\partial \ell (\Theta )}{\partial \xi }$ to zero, and simultaneously solving these expressions, yields the MLEs $\hat{\theta }$ and $\hat{\xi }$, respectively.

### Monte Carlo simulation study

In this sub-section, we investigate the performance of the MLEs. For the simulation purposes, the special sub-model TI-HTW distribution is considered. The simulation process is conducted based on the following steps:

- *N* = 1000 samples of size *n* = 25, 50, 75, …, 1000 are generated from TI-HTW model with parameters *α*, *γ* and *θ*. The inversion procedure of generating random number is used.
- Compute MLEs of (*α*, *γ*, *θ*).
- Compute biases and mean square error (MSE) of the model parameters.
- Coverage probabilities (CPs) are calculated at the 95% confidence interval (C.I).
- Steps (i)-(iii) are repeated for *n*.

The simulation results are provided in Tables 3 and 4. The results in these tables indicate that the behavior of the estimates of the TI-HTW parameters are good, showing small bias and creditable MSEs in all studied cases; that is, these estimates are quite reliable and very close to the actual values. Further, the biases are approaching to 0 as the sample size increases, proving that the estimates are behaved asymptotically unbiased estimators. Moreover, the MSEs decrease as the sample size increases, showing that these estimators are consistent for the TI-HTW parameters.

**Table 3:** Simulation results for different combination of the parameters of TI-HTW distribution.

Set 1: *α* = 0.8, *θ* = 0.5, *γ* = 1,							
*n*	Par	MLE	Biases	MSE	C.I		CPs
25	*α*	0.9058	0.1058	0.0464	(0.4925	1.3190)	0.961
	*θ*	0.7020	0.2020	0.2934	(-1.2089	2.6131)	0.847
	*γ*	1.3591	0.3591	2.4261	(-2.9500	5.6683)	0.979
100	*α*	0.8440	0.0440	0.0154	(0.6061	1.0818)	0.902
	*θ*	0.6534	0.1534	0.2310	(-0.4883	1.7952)	0.866
	*γ*	1.2041	0.2041	1.1954	(-1.1441	3.5524)	0.957
300	*α*	0.8229	0.0229	0.0079	(0.6612	0.9846)	0.859
	*θ*	0.5894	0.0894	0.1275	(-0.0456	1.2244)	0.882
	*γ*	1.1141	0.1141	0.5440	(-0.1608	2.3891)	0.944
600	*α*	0.8211	0.0211	0.0056	(0.7001	0.9420)	0.872
	*θ*	0.5732	0.0732	0.0658	(0.1417	1.0047)	0.912
	*γ*	1.0071	0.0071	0.2362	(0.1502	1.8641)	0.957
900	*α*	0.8123	0.0123	0.0037	(0.7083	0.9163)	0.885
	*θ*	0.5465	0.0465	0.0438	(0.2002	0.8929)	0.881
	*γ*	1.0165	0.0165	0.1641	(0.2694	1.7636)	0.958
1000	*α*	0.8172	0.0172	0.0043	(0.7142	0.9201)	0.897
	*θ*	0.5364	0.0664	0.0528	(0.2069	0.9259)	0.892
	*γ*	0.9938	-0.0161	0.1706	(0.2754	1.6922)	0.940

**Table 4:** Simulation results for different combination of the parameters of TI-HTW distribution.

Set 1: *α* = 1.4, *θ* = 0.9, *γ* = 1,							
*n*	Par	MLE	Biases	MSE	C.I		CPs
25	*α*	1.5213	0.1213	0.0912	(0.8441	2.1986)	0.961
	*θ*	1.1296	0.3296	0.6663	(-2.1895	4.4488)	0.921
	*γ*	1.1683	0.1683	1.3508	(2.2242	4.5610)	0.915
100	*α*	1.4384	0.0384	0.0378	(1.0422	1.8347)	0.896
	*θ*	1.0504	0.2504	0.5678	(1.0994	3.2004)	0.905
	*γ*	1.1733	0.1733	1.0224	(-1.0550	3.4017)	0.907
300	*α*	1.4253	0.0253	0.0214	(1.1451	1.7054)	0.849
	*θ*	0.9819	0.1819	0.3795	(-0.3003	2.2641)	0.894
	*γ*	1.0921	0.0921	0.5835	(-0.3796	2.5639)	0.890
600	*α*	1.4115	0.0115	0.0137	(1.1836	1.6393)	0.856
	*θ*	0.9607	0.0907	0.1747	(0.1257	1.6557)	0.903
	*γ*	1.0877	0.0877	0.3805	(-0.0901	2.2657)	0.915
900	*α*	1.4011	0.0011	0.0116	(1.2065	1.5957)	0.854
	*θ*	0.8458	0.0458	0.1028	(0.2963	1.3953)	0.883
	*γ*	1.1093	0.0843	0.3304	(0.0925	2.1262)	0.916
1000	*α*	1.4063	0.0006	0.0103	(1.2203	1.5923)	0.851
	*θ*	0.8771	0.0421	0.0870	(0.3210	1.3931)	0.896
	*γ*	1.0746	0.0746	0.2966	(0.12839	2.0208)	0.906

## Actuarial measures

In actuarial sciences and management institutions, one of the key tasks of the actuaries is to evaluate the exposure of market risk in a portfolio of instruments. In this section, we calculate some important risk measures including value at risk (VaR) and tail value at risk (TVaR) for the TI-HTW, which play a crucial role in portfolio optimization under uncertainty.

### VaR measure

Let *X* follow the TI-HTW model with pdf (7), then the VaR of *X* denoted by VaR_*q*_ (*q* is a specified level of significance) is given by

VaRq=xq=(−1γlog{1−((1−q)1θ−1(1−θ)(1−q)1θ−1)})1α. (20)

### TVaR measure

The TVaR is one of the most important risk measures that quantifies the expected loss provided that an event outside a specified level of probability has occurred. Let *X* has the TI-HTW model, then the TVaR of *X* is computed as

$$TVaR(X)=\int_{VaR_{q}}^{\infty }xg(x;\theta ,\xi )dx,$$

$$TVaR(X)=\frac{\theta^{2}}{1-q}\int_{VaR_{q}}^{\infty }x\frac{f(x;\xi ){\left\{1-F(x;\xi )\right\}}^{\theta -1}}{{\left\{1-(1-\theta )F(x;\xi )\right\}}^{\theta +1}}dx.$$ (21)

Inserting (12) and (14) in (21), we get

$$\begin{array}{c} TVaR(X)=\frac{A_{i,j,\theta }}{1-q}\int_{VaR_{q}}^{\infty }xf(x;\xi )(F(x;\xi ){)}^{i+j}dx, \end{array}$$ (22)

where $A_{i,j,\theta }=\theta^{2}\sum_{i=0}^{\infty }\sum_{j=0}^{\theta -1}(\begin{array}{c} \theta -1\\ j \end{array})(\begin{array}{c} i+\theta \\ \theta \end{array})(-1{)}^{j}(1-\theta {)}^{i}.$

On solving we get

$$\begin{array}{c} TVaR(X)=B_{i,j,k,\theta }\Gamma (\frac{1}{\alpha }+1,\gamma (k+1)(VaR_{q}{)}^{\alpha }), \end{array}$$ (23)

where $B_{i,j,k,\theta }=\theta^{2}\sum_{i=0}^{\infty }\sum_{j=0}^{\theta -1}\sum_{k=0}^{i+j}\left(-1{)}^{j+k}(1-\theta {)}^{i}(\begin{array}{c} \theta -1\\ j \end{array})(\begin{array}{c} i+\theta \\ \theta \end{array})(\begin{array}{c} i+j\\ k \end{array}\right).$

### Numerical study of the risk measures

In the current sub-section, we conduct numerical study of the VaR and TVaR measures for the TI-HTW distribution. The VaR and TVaR of the TI-HTW distribution are compared with the Weibull distribution as a nested model and the exponentiated Weibull (EW) distribution [24] as a non-nested model, which is one the most prominent generalization of the Weibull model. The numerical results are obtained as follows.

**Fig 5:**
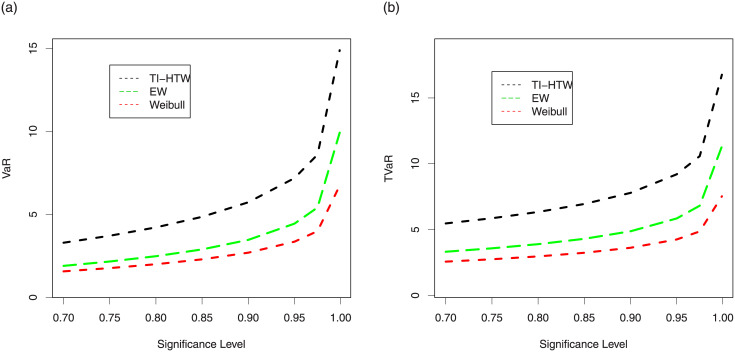
Graphical display of the results provided in Table 5.

**Fig 6:**
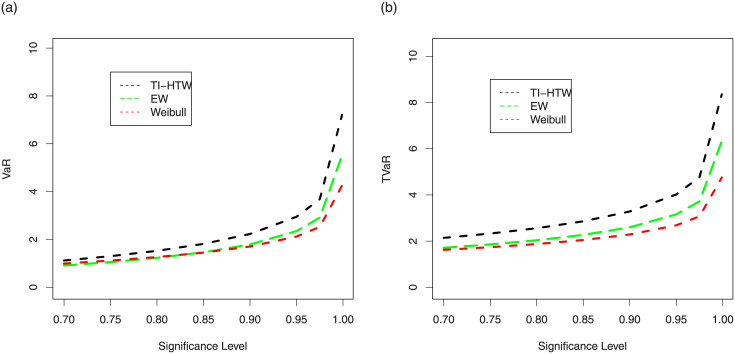
Graphical display of the results provided in Table 6.

1. We generated a sample of size *n* = 100 from the Weibull, EW and TI-HTW distributions and their parameters have been estimated via ML method.
2. 1000 repetitions are made to calculate the VaR and TVaR for these distributions.
3. The numerical results of the risk measures are provided in Tables 5 and 6. Further, these results are displayed graphically in Figs 5 and 6, respectively.

**Table 5:** Simulation results for VaR and TVaR of the Weibull, EW and TI-HLW distributions.

Dist.	Parameters	Level of significance	VaR	TVaR
Weibull		0.700	1.5601	2.5514
		0.750	1.7546	2.7307
	$\hat{\alpha }=1.2$	0.800	1.9870	2.9467
	$\hat{\gamma }=0.7$	0.850	2.2788	3.2200
		0.900	2.6780	3.5971
		0.950	3.3346	4.2231
		0.975	3.9662	4.8302
		0.999	6.6897	7.4798
EW		0.700	1.8921	3.3055
		0.750	2.1533	3.5628
	$\hat{\alpha }=1.2$	0.800	2.4710	3.8769
	$\hat{\theta }=0.9$	0.850	2.8784	4.2809
	$\hat{\gamma }=0.7$	0.900	3.4496	4.8488
		0.950	4.4214	5.8174
		0.975	5.3900	6.7845
		0.999	9.8769	11.2700
TI-HTW		0.700	2.9383	5.0410
		0.750	3.3356	5.4229
		0.800	3.8153	5.8869
	$\hat{\alpha }=1.2$	0.850	4.4255	6.4804
	$\hat{\theta }=0.9$	0.900	5.2735	7.3096
	$\hat{\gamma }=0.7$	0.950	6.7000	8.7122
		0.975	8.1061	10.1003
		0.999	14.4855	16.4291

**Table 6:** Simulation results for VaR and TVaR of the fWeibull, EW and TI-HLW distributions.

Dist.	Parameters	Level of significance	VaR	TVaR
Weibull		0.700	0.9927	1.6235
		0.750	1.1165	1.7377
	$\hat{\alpha }=0.9$	0.800	1.2644	1.8751
	$\hat{\gamma }=1.2$	0.850	1.4501	2.0490
		0.900	1.7041	2.2889
		0.950	2.1219	2.6873
		0.975	2.5238	3.0736
		0.999	4.2569	4.7597
EW		0.700	0.9062	1.7096
		0.750	1.0514	1.8562
	$\hat{\alpha }=0.9$	0.800	1.2297	2.0359
	$\hat{\theta }=1.5$	0.850	1.4605	2.2681
	$\hat{\gamma }=1.2$	0.900	1.7870	2.5958
		0.950	2.3469	3.1569
		0.975	2.9080	3.7186
		0.999	5.5179	6.3291
TI-HTW		0.700	1.1235	2.1527
		0.750	1.3052	2.3409
		0.800	1.5298	2.5728
	$\hat{\alpha }=0.9$	0.850	1.8223	2.8742
	$\hat{\theta }=1.5$	0.900	2.2398	3.3030
	$\hat{\gamma }=1.2$	0.950	2.9657	4.0460
		0.975	3.7049	4.8001
		0.999	7.2677	8.4141

The simulation is performed for the Weibull, EW and proposed models for selected values of their parameters. A model with higher values for VaR and TVaR is said to have a heavier tail. The simulated results provided in Tables 5 and 6 shows that the proposed TI-HTW model has higher values of the risk measures than the Weibull and EW distributions. Figs 5 and 6 also show that the proposed model has a heavier tail than the Weibull and EW distributions.

## Comparative study

This section, we consider three heavy-tailed data from applied areas such as medical, engineering and financial sciences to study the flexibility of the proposed family. The key motivations of considering the heavy-tailed distributions are that they adequately provide the best fit to the heavy-tailed data. For each data set, the TI-HTW distribution is compared with different well-known distributions and we observed that the proposed distribution outclass other competitors.

To decide about the goodness of fit among the applied distributions, we consider certain analytical measures. In this regard, we consider two discrimination measures such as the Akaike information criterion (AIC) and Bayesian information criterion (BIC); see [25].

In addition to the discrimination measures, other goodness of fit measures such as Cramer-Von-Mises (CM) test statistic, Anderson Darling (AD) test statistic and Kolmogorov–Smirnov (KS) test along with its p-values are also considered. The formulae for these measures can be found in [26].

A distribution with lower values of these analytical measures is considered to be a good candidate model among the applied distributions for the underlying data sets. By considering these statistical tools, we observed that the TI-HTW model is the best competitor compared to other models because the values of all selected criteria are significantly small for it.

### A real life application from bio-medical sciences

The first data set is reported in [27], and it refers to the remission times of bladder cancer patients. For the first data set, the TI-HTW distribution is compared with the Weibull, MOW [28], MW [29], TW [30] and APTW [31] distributions. A number of authors have been used these distributions to model bio-medical data sets. For example, [32] used the Weibull and MOW distributions to model the survival times of the cancer patients. These data were analyzed by [33] and [34].

**Fig 7:**
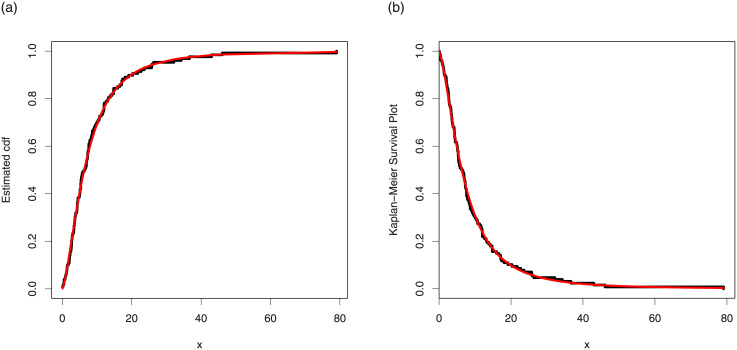
Estimated cdf and Kaplan Meier survival plots of the TI-HTW distribution for data 1.

**Fig 8:**
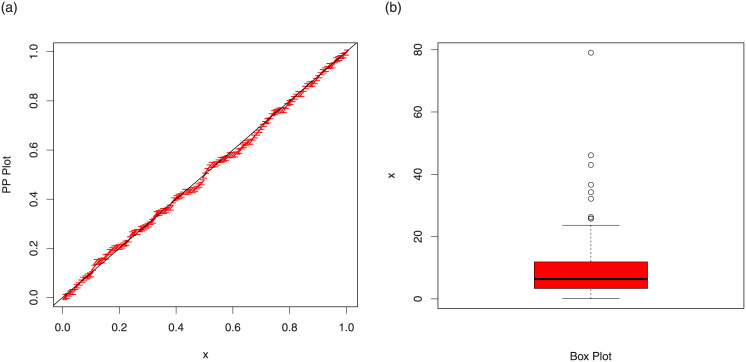
PP plot of the TI-HTW distribution and the box plot for data 1.

The maximum likelihood estimates of the models for cancer data are presented in Table 7. The analytical measures of the competitive models are provided in Table 8. Form Table 8, it is clear that the proposed distribution has lower values of these measures than the other models. The fitted cdf and Kaplan-Meier survival plots of the proposed model for cancer data are plotted in Fig 7. The PP plot of the TI-HTW model and box plot of the cancer data are sketched in Fig 8. From Fig 7, we can see that the proposed model fits the estimated cdf and Kaplan Meier survival plots very closely. From Fig 8, we can easily detect that the data set is skewed to the right (see box plot) and proposed model is closely followed the PP-plot.

**Table 7:** Estimated values with standard error (in parenthesis) of the competitive models for data 1.

*Dist*.	*α*	*γ*	*σ*	λ	*α*_1_	*θ*
TI-HTW	1.4273 (0.1774)	0.028 (0.0112)				2.0711 (0.9573)
Weibull	1.047 (0.0675)	0.093 (0.0190)				
APTW	0.014 (0.0865)	0.016 (0.0064)			0.014 (0.0216)	
MOW	1.268 (0.1308)	0.877 (0.5205)	11.829 (11.2869)			
TW	1.133 (0.0753)	0.047 (0.0113)		0.744 (0.2021)		
MW	1.007 (0.0313)	0.951 (4.2501)				0.863 (4.2551)

**Table 8:** Discrimination and goodness of fit measures of the TI-HTW and other competitive models for data 1.

*Dist*.	AIC	BIC	CM	AD	KS	p-value
TI-HTW	825.479	834.035	0.019	0.130	0.035	0.997
Weibull	832.173	837.877	0.131	0.786	0.069	0.558
APTW	826.378	836.934	0.042	0.255	0.045	0.949
MOW	834.988	843.544	0.150	0.884	0.075	0.451
TW	829.916	838.472	0.086	0.516	0.058	0.768
MW	833.969	842.525	0.133	0.797	0.073	0.494

### A real life application from reliability engineering

Here, we investigate the TI-HTW distribution via analyzing a heavy-tailed reliability engineering data which are reported in [35], and they refer to failure time of coating machine. To show the potentiality of the proposed method, the TI-HTW distribution is applied in comparison with the Ex-APTW, Ku-W and BW distributions. The Ku-W [36]and Ex-APTW [37] have been used to model failure times data. Al-Malki [38] showed that the BW distribution is one of the most prominent extensions of the Weibull distribution that can be used quite effectively in failure rate time data.

**Fig 9:**
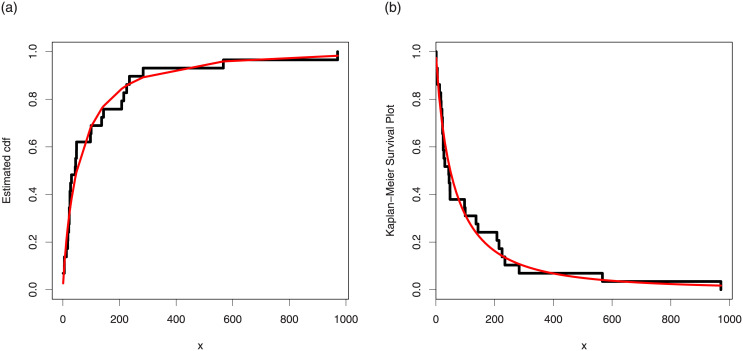
Estimated cdf and Kaplan Meier survival plots of the TI-HTW distribution for data 2.

**Fig 10:**
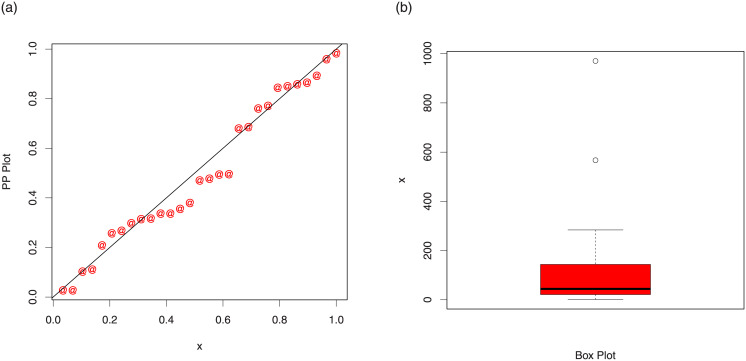
PP plot of the TI-HTW distribution and the box plot for data 2.

Corresponding to data set 2, the values of the model parameters are reported in Table 9. The analytical measures of the proposed and other competitive models are provided in Table 10. The estimated cdf and Kaplan-Meier survival plots are sketched in Fig 9, which show that proposed distribution fits the estimated cdf and Kaplan-Meier survival plots very closely. The PP and box plots are sketched in Fig 10.

**Table 9:** Estimated values of the model parameters with standard error (in parenthesis) of the fitted models for data 2.

*Dist*.	*α*	*γ*	*θ*	*α*_1_	*a*	*b*
TI-HTW	0.525 (0.0746)	0.843 (0.5898)	0.149 (0.1097)			
Ex-APTW	0.510 (0.5094)	0.172 (0.6258)		5.425 (7.0766)		
Ku-W	0.620 (0.3093)	0.501 (1.0970)			0.702 (3.2715)	0.118 (2.0964)
BW	0.478 (0.2696)	0.502 (0.5522)			2.797 (3.1595)	0.344 (0.6646)

**Table 10:** Analytical measures of of the TI-HTW and competitive models for data 2.

*Dist*.	AIC	BIC	CM	AD	KS	p-value
TI-HTW	333.949	337.050	0.060	0.336	0.124	0.756
Ex-APTW	335.071	339.172	0.093	0.491	0.142	0.598
Ku-W	337.750	343.220	0.091	0.546	0.146	0.488
BW	335.457	340.926	NaN	NaN	0.144	0.603

### A real life application from insurance sciences

The third data set from the insurance sciences and represents the vehicle insurance losses which are available at: http://www.businessandeconomics.mq.edu.au. For the third data, the TI-HTW distribution is compared with the Weibull, Lomax and Burr-XII distributions which are widely used in modeling financial and financial risk management problems. The Weibull distribution is one of the best competitors for modeling actuarial data up to a specified threshold; see [39]. Further, the Lomax [40] and Burr [41] distributions have been widely used in data modeling with tail beyond the threshold.

**Fig 11:**
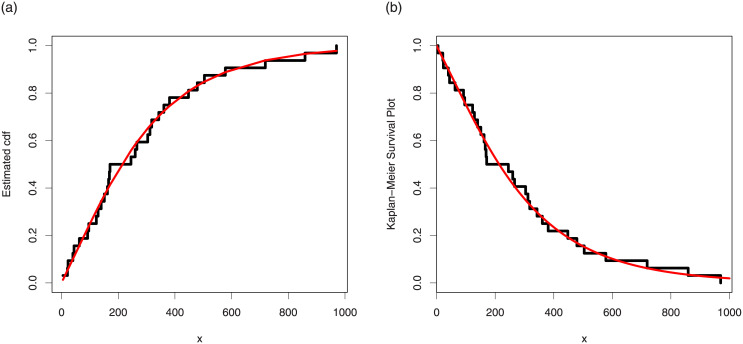
Estimated cdf and Kaplan Meier survival plots of the TI-HTW distribution for data 3.

**Fig 12:**
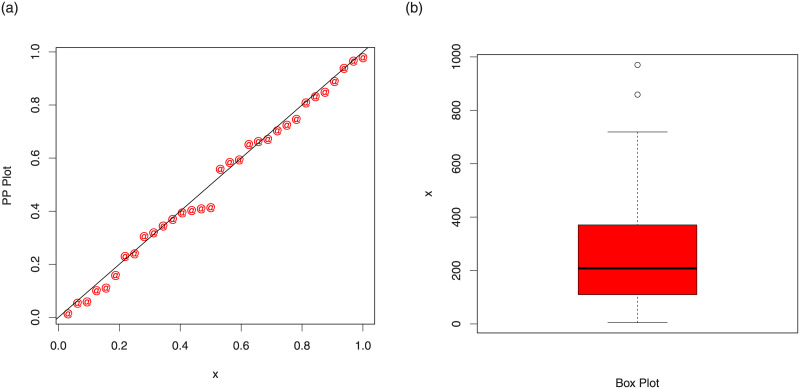
PP plot of the TI-HTW distribution and the box plot for data 3.

The parameter values are reported in Table 11 for the insurance data, and the analytical measures are presented in Table 12. The estimated cdf and Kaplan-Meier survival plots are sketched in Fig 11. The PP and box plots are sketched in Fig 12. From Figs 11 and 12, it is clear that the proposed model fits the estimated cdf, Kaplan-Meier survival and PP plots very well.

**Table 11:** Maximum likelihood estimates of the fitted models with standard error (in parenthesis) for data 3.

Dist.	$\hat{\alpha }$	$\hat{\gamma }$	$\hat{\theta }$	$\hat{c}$	$\hat{k}$
TI-HTW	0.955 (0.1304)	0.012 (0.0145)	0.483 (0.2849)		
Weibull	1.019 (0.9445)	0.003 (1.6540)			
Lomax	0.495 (0.4334)	30.008 (9.6185)			
Burr				0.049 (0.1134)	4.427 (2.2671)

**Table 12:** Analytical measures of the TI-HTW and other competing models for data 3.

*Dist*.	AIC	BIC	CM	AD	KS	p-value
TI-HTW	429.743	434.140	0.021	0.131	0.086	0.951
Weibull	432.353	439.256	0.054	0.447	0.185	0.597
Lomax	460.191	463.021	0.083	0.520	0.207	0.108
Burr	503.477	506.383	0.228	1.362	0.416	0.208

## Baysesian analysis

We adopt a Bayesian formulation for our proposed model and drive posterior inference using Markov chain Monte Carlo (MCMC) algorithm. To generate MCMC samples from posterior distribution of the parameters of our joint model for Bayesian inference, we have used WinBUGS software [42]. Note that to specify a likelihood contribution for a distribution that is not listed in WinBUGS, we have used the “zeros trick” [43]. In particular, a Poisson (λ) observation of zero has likelihood exp(−λ), so if our observed data is set of 0’s, and λ[*i*] is set to −log(*L*[*i*]), we obtain the correct likelihood contribution. Since, λ[*i*] should always be >0 as it is a Poisson mean, we may need to add suitable constant to ensure that is positive. This is equivalent to multiplying each likelihood term by *e*^−*c*^. This process does not influence the likelihood since it is equivalent to multiplying the resulting posterior distribution by a constant term equal to *e*^−*nc*^. Thus, the likelihood takes the form

$$\begin{array}{c} f\left(t|\theta \right)=\prod_{i=1}^{n}\frac{e^{-(-\ell_{i}+C)}{\left(-\ell_{i}+C\right)}^{0}}{0!}=\prod_{i=1}^{n}f_{p}\left(0;-l_{i}+C\right). \end{array}$$ (24)

The choice of a good prior distribution plays a key role in Bayesian inference. In practice, no information is precise enough to lead to the exact determination of the prior distribution. However, non-informative prior that allows the data to dominate to determine the posterior distribution are suggested for the Bayes-MCMC methods. We consider standard distribution for priors, such as gamma priors for *α*, *γ* and *θ*, as these are positive-valued random variables. Note that gamma priors are widely used in Bayesian literature for positive-valued random variables. For assessing convergence, a simple (informal) method of assessing chain convergence is to look at some graphical diagnostics such as trace plot, autocorrelation plot and density plots to determine the mixing of chains. If the chains show a reasonable degree of randomness between iterations, it signifies that the Markov chain has found an area of high likelihood and is integrating over the target density and hence indicating that it has converged. Moreover, we also use the Gelman-Rubin statistic R, another popular technique for diagnosing convergence. It is based on comparison of with in chain and between chain variances. Values of R substantially above 1 indicate lack of convergence. However, some authors suggests that *R* <1.2 is acceptable. To examine the empirical performance of the proposed methodology for model adequacy, deviance information criterion (DIC) is the most widely used criterion for model comparison in Bayesian analysis [44] and [45]. It is derived based on two principles: (i) goodness of fit measured via the deviance statistic, and (ii) model complexity measured by an estimate of the effective number of parameters, denoted by *p*_*D*_. When comparing two or more models, it is suggested that *DIC*_*M*_ − *DIC*_*min*_ > 10 or if the difference lies between 5 and 10, then there is considerably less support for Model M compared to the model with minimum DIC. However, *DIC*_*M*_ − *DIC*_*min*_ < 5 shows that no support for a model with the lowest DIC and may lead to misleading inference.

### A real life application to AIDS data

[46] described a study involving 467 human immunodeficiency virus (HIV) infected patients who had failed or were intolerant to zidovudine therapy (ZT). The main objective was to compare two antiretroviral drugs to prevent the progression of HIV infections: didanosine (ddI) and zalcitabine (ddC). To analyze the data, We construct two Morkov chains each of 100,000 iteration to approximate posterior density, each following a 10,000 iteration as a burn-in period. we consider here only the TI-HTW and Weibull distributions to model the time-to-event process. For sake of simplicity, we summarize only DIC values and distributional parameters values in Table 13.

**Table 13:** Bayesian analysis of the TI-HTW and Weibull models.

Parameter	TI-HTW PH (DIC = 7314.9)			Weibull PH (DIC = 7314.9)		
	Posterior Median	Standard Deviation	95% Credible Interval	Posterior Median	Standard Deviation	95% Credible Interval
*α*	0.003	0.004	0.002, 0.007	0.002	0.001	0.003, 0.005
*γ*	1.158	0.143	1.116, 1.655	1.492	0.099	1.305, 1.694
*θ*	1.902	0.399	0.774, 2.484			

DIC values of the TI-HTW distribution and Weibull fits are 7314.9 and 7328.83, respectively, suggesting that the TI-HTW distribution has a superior fit over the Weibull distribution.

## Conclusions

The importance of the extended distributions first realized in financial sciences and later in other applied fields such as engineering and medical sciences. To cater data in those fields, a number of methods have been introduced. In this context, we have studied a versatile three parameters heavy-tailed model, called type-1 heavy tailed Weibull distribution as a special case of a new approach allowing closed form expressions for some basic mathematical and other related properties. The proposed class is called type-I heavy-tailed family. The usefulness of the proposed family of heavy-tailed distributions has been proved via three data sets from medical, engineering and financial sciences and the model performs reasonably good than the well-known competing heavy-tailed distributions. The developed family in this work is a promising method for modeling data in the distribution theory, may be useful for the researchers who deal with such data sets. Thus, the new model can be served as a good competitor alternative to other existing models.

Future work includes (i) bivariate extension of the actuarial measures and the Monte Carlo simulation study of these measures, (ii) modeling heavy-tailed data with bivariate extension, (iii) regression problems with covariates and (iv) parameter reduction.
